# Supplementary material for: Polymer Dispersed Cholesteric Liquid Crystals with a Toroidal Director Configuration under an Electric Field
Source: Polymers (Basel). 2021 Feb 27;13(5):732. doi: 10.3390/polym13050732 (PMC7956821; doi:10.3390/polym13050732)
Supplement: Supplementary file 1 [file polymers-13-00732-s001.zip › MDPI_Polymers_PDCLC_SI.pdf]

Supplementary Materials: Polymer Dispersed Cholesteric Liquid Crystals With a Toroidal Director Configuration Under an Electric Field

Anna P. Gardymova [1], Mikhail N. Krakhalev [1,2], Victor Ya. Zyryanov [2], Alexandra A. Gruzdenko [3], Andrey A. Alekseev [3] and Vladimir Yu. Rudyak [3]*

1 S1. Experimental dependencies of the a/d ratio on the applied electric field E

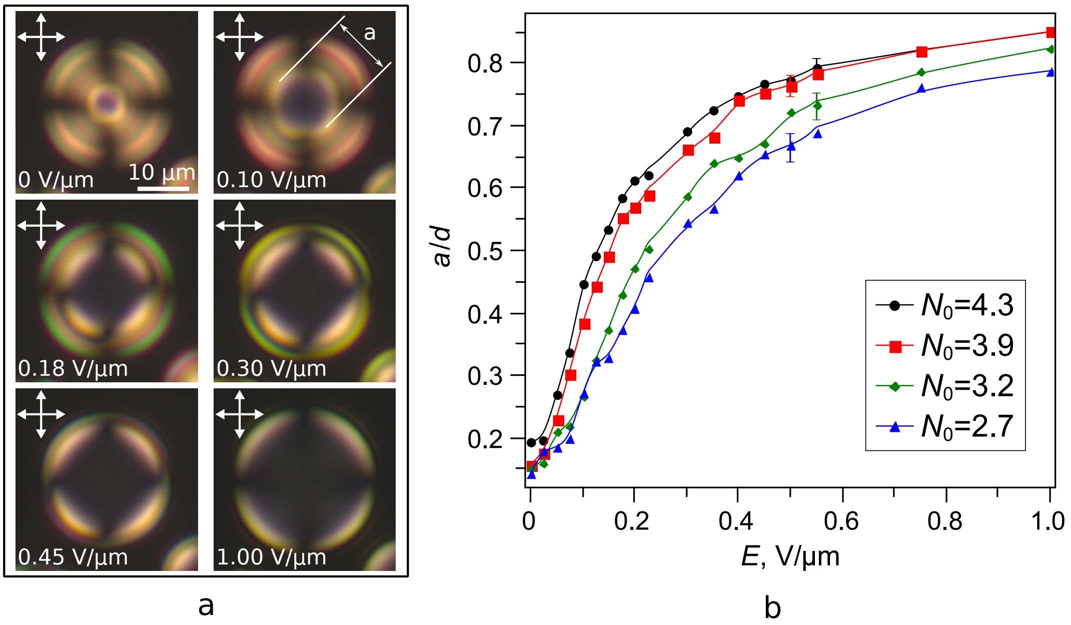

Figure S1. POM photos of the cholesteric droplet at $N_0 = 3.9$ taken in the crossed polarisers for different applied electric fields values (a). Dependencies of the a/d ratio on the applied electric field E obtained for CLC droplets at different values of N_0. Intrinsic helix pitch is $p_0 = 14$ μm.

2 S2. Calculated dependencies of the a/d ratio on the applied dimentionless electric field e

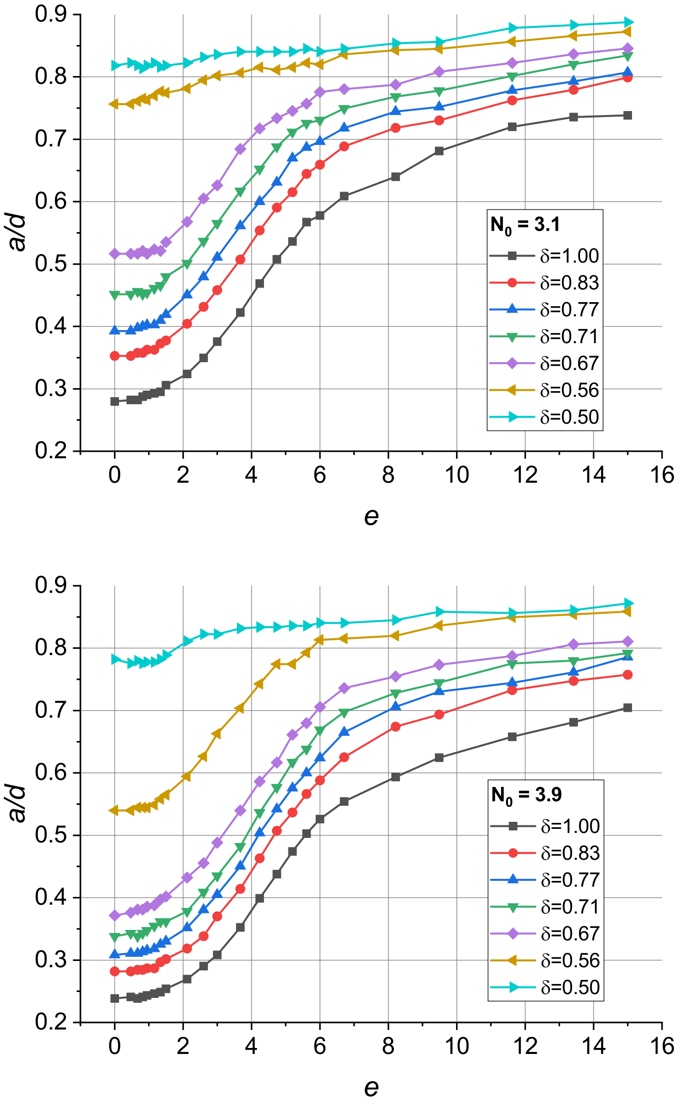

Figure S2. Calculated dependencies $a/d(e)$ for chiral parameter $N_0 = 3.1$ and 3.9 in droplets of various oblateness δ.
